# Discrimination and Quantification of Cotton and Polyester Textile Samples Using Near-Infrared and Mid-Infrared Spectroscopies

**DOI:** 10.3390/molecules29153667

**Published:** 2024-08-02

**Authors:** Maria Luís Paz, Clara Sousa

**Affiliations:** CBQF–Centro de Biotecnologia e Química Fina–Laboratório Associado, Escola Superior de Biotecnologia, Universidade Católica Portuguesa, Rua Diogo Botelho 1327, 4169-005 Porto, Portugal; marialuispaz@gmail.com

**Keywords:** cotton, polyester, chemometrics, PLS models, model validation

## Abstract

In the textile industry, cotton and polyester (PES) are among the most used fibres to produce clothes. The correct identification and accurate composition estimate of fibres are mandatory, and environmentally friendly and precise techniques are welcome. In this context, the use of near-infrared (NIR) and mid-infrared (MIR) spectroscopies to distinguish between cotton and PES samples and further estimate the cotton content of blended samples were evaluated. Infrared spectra were acquired and modelled through diverse chemometric models: principal component analysis; partial least squares discriminant analysis; and partial least squares (PLS) regression. Both techniques (NIR and MIR) presented good potential for cotton and PES sample discrimination, although the results obtained with NIR spectroscopy were slightly better. Regarding cotton content estimates, the calibration errors of the PLS models were 3.3% and 6.5% for NIR and MIR spectroscopy, respectively. The PLS models were validated with two different sets of samples: prediction set 1, containing blended cotton + PES samples (like those used in the calibration step), and prediction set 2, containing cotton + PES + distinct fibre samples. Prediction set 2 was included to address one of the biggest known drawbacks of such chemometric models, which is the prediction of sample types that are not used in the calibration. Despite the poorer results obtained for prediction set 2, all the errors were lower than 8%, proving the suitability of the techniques for cotton content estimation. It should be stressed that the textile samples used in this work came from different geographic origins (cotton) and were of distinct presentations (raw, yarn, knitted/woven fabric), which strengthens our findings.

## 1. Introduction

The textile industry is known as one of the most polluting manufacturing fields globally. As of late, with the ongoing efforts to reduce the environmental impact of businesses, this industry is facing some challenges, and all attempts to minimize CO_2_ emissions, such as the use of greener techniques for sample identification and characterization, should be considered. Spectroscopic techniques, particularly near-infrared (NIR) and mid-infrared (MIR) spectroscopy, are lauded for their minimal carbon footprint, rapid analysis time, cost-effectiveness, versatility, and result accuracy [1,2]. These advantages have led to their widespread implementation in characterizing samples from diverse origins [3], including those within the textile industry [4,5]. 

Cotton reigns supreme as the most significant natural fibre in the textile industry. Its dominance can be attributed to the remarkable properties it displays, making this fibre ideal for clothing and domestic fabrics. Cotton boasts inherent strength, breathability, and a soft and comfortable feel due to its unique fibre structure [6]. Cotton’s natural absorbency also makes this fibre ideal for undergarments and sportswear [7] and, considering its hypoallergenic properties, it is a popular choice for individuals with sensitive skin. These characteristics, coupled with cotton’s long history of cultivation and processing, ensures its continued relevance in the textile industry. On the other hand, polyester (PES) is one of the most popular man-made fibres. Its dominance can be attributed to its impressive strength, shape retention, and durability, making it ideal for long-lasting garments and industrial applications [8,9]. PES’s versatility allows it to be blended with natural fibres like cotton, offering breathability and comfort while enhancing properties like wrinkle resistance [10]. 

Some attempts to characterize textile samples made of cotton and/or PES (pure or blended) through infrared spectroscopy have been reported in the literature. Regarding MIR spectroscopy, Peets and colleagues (2017) made a successful attempt to classify pure and binary mixtures of several textile fibres, including cotton and polyester, among others [11]. Later, Peets et al. (2019) compared ATR-FTIR (attenuated total reflectance Fourier transform infrared) spectroscopy, microATR-FTIR, and reflectance FTIR to discriminate single-component fibres [12]. Riba and co-workers successfully tested a data fusion technique to classify textile waste [13]. Also, NIR spectroscopy is regarded as a useful and reliable technique for cotton and PES sample characterization [14,15,16] and content estimates [17,18,19]. Cleve and colleagues [14] used NIR spectroscopy to discriminate pure (100%) cotton, PES, silk, wool, acetate, and polyacrylonitrile fibres, while Zhou and co-workers used this technique for fibre identification (including pure samples) in the context of textile recycling [15]. Sun et al. (2016) [16] also included one cotton + polyester blended sample. NIR spectroscopy was also employed for quantification proposes [17,18,19], with quite promising results. In addition, hyperspectral near-infrared imaging spectroscopy, coupled with machine learning techniques, was reported as a successful technique to estimate the polyester content of blended samples [20]. Despite some of the published literature in the field, the great majority of the works only consider a single type of samples (knitted and/or woven fabric) for discrimination proposes. Regarding composition estimates, no attempts using MIR spectroscopy have been found. Among NIR studies, only a few included validation samples, which were of the same type as the ones used to calibrate the models. To the best of our knowledge, no attempts to validate chemometric models with distinct sample types (including different fibres) have been reported.

In this work, the NIR and MIR signatures of cotton and PES (pure and blended) samples (raw, yarn, and knitted and woven fabric) were analyzed by principal component analysis (PCA) and partial least squares discriminant analysis (PLSDA). Further, they were used to estimate their cotton contents through a partial least squares (PLS) regression analysis. PLS models were optimized and further tested with two distinct and totally independent prediction sets of samples: blended cotton/polyester samples (like those used to develop the PLS model) and additional blended samples containing cotton, PES, and different types of fibre (linen, viscose, elastane, and polyamide). 

## 2. Results

### 2.1. Spectral Overview

**Figure 1:**
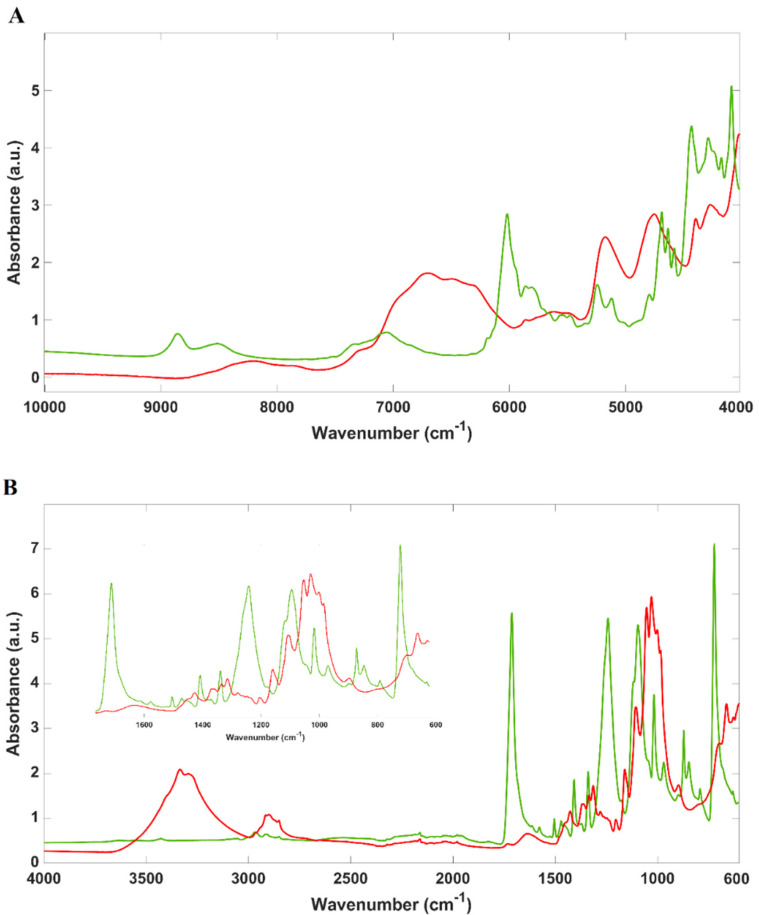
(**A**) Near-infrared and (**B**) mid-infrared spectra (mean spectra of all the samples) of 100% cotton and 100% polyester samples. Legend: red lines—mean spectrum of cotton samples; green lines—mean spectrum of PES samples.

Near-infrared and mid-infrared spectra of 100% cotton and 100% PES (mean spectrum of all the samples of each fibre) are shown in Figure 1A,B, respectively.

It is well known that cotton fibres are mostly composed of cellulose (88.0–96.0%), possessing other constituents, such as proteins (1.1–1.9%), pectic substances (0.7–1.2%), wax (0.4–1.0%), and sugars (~0.3%), among others, in a much lower concentration [21]. Due to the elevated cellulose content, the vibrational spectra obtained from cotton fibres will be dominated by cellulose vibrations. Near-infrared spectra (10,000–4000 cm^−1^) reflect the overtones (third, 14,000–8695 cm^−1^; second, 9524–6060 cm^−1^; and first, 6780–4878 cm^−1^) and combination bands (5128–4000 cm^−1^) of the R-H groups (such as C-H, N-H, and O-H, among others) present in the samples. Bands from water can be observed in NIR spectra and reflect the samples’ humidity. Water bands are mainly located at 5250 cm^−1^ (combinations of O-H stretching and deformation) and 5150 cm^−1^ (O-H stretching) and between 7000 and 6800 cm^−1^. As expected, cotton samples globally present a higher water content (higher intensity of water bands—Figure 1A) than PES samples. Cotton, due to its cellulosic nature, possesses a high number of alcohol groups, which increase the degree of moisture penetration. Besides water, near-infrared spectra of cotton possess vibrations at 8230–8000 cm^−1^ associated with the second overtone of the carbon–hydrogen–oxygen stretching vibration in cellulose [5] and at 6712 cm^−1^ and 4739 cm^−1^ (both from the combined O-H stretching and bending vibrations) [22]. On the other hand, PES is a synthetic fibre derived from the polycondensation of organic diacid and dihydric alcohol, retaining typical carbonyl bonds and methylene groups, among others. Near-infrared PES spectra display vibration bands at 6100–6000 cm^−1^, corresponding to the first overtone of -CH and -CH_2_ vibrations, and at around 5800 cm^−1^, related to the second overtone of the carbonyl bond [23,24].

Regarding the mid-infrared spectra of cotton and PES, typical bands reflecting the main constituents of both samples can be observed. Cotton presents typical -OH stretching vibrations at 3450–3340 cm^−1^, reflecting the sample’s water content [25], and vibrations between 2900 and 2800 cm^−1^, depicting the -CH stretching vibrations [26] of cellulose (2925 cm^−1^—asymmetric and 2865 cm^−1^—symmetric). In the region from 1450 to 1310 cm^−1^, cotton spectra also render vibrations associated with C-H bending in cellulose [26], and at 1145–1155 cm^−1^, ones associated with C-O-C and C-H stretching, also from the structure of cellulose [26]. PES possesses a typical spectrum with a key difference from cotton: the absence of the strong O-H stretching band at around 3450–3340 cm⁻^1^ (due to the presence of cellulose and adsorbed water). This band is absent in the mid-infrared spectrum of PES because it lacks hydroxyl groups. PES spectra also exhibit a strong vibration band between 1725 and 1715 cm^−1^, due to the vibration of the carbonyl group (C=O) [27]. Other vibrations can be observed, namely at 1270–1240 cm^−1^ and 1120–1100 cm^−1^ (C-O-C stretching of the ester groups), at 980–970 cm^−1^ (C-H deformation in aromatic rings), and at 800–700 cm^−1^ (out-of-plane C-H bending) [27].

### 2.2. Cotton/Polyester Sample Discrimination

**Figure 2:**
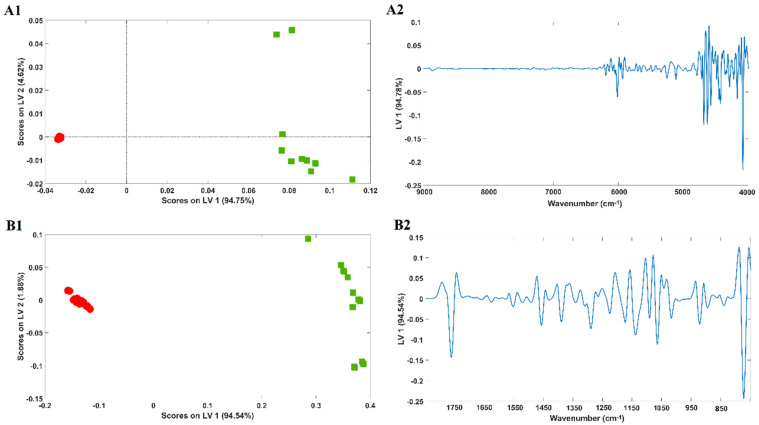
Score plots of the PLSDA regression models obtained for 100% cotton and 100% PES samples. (**A1**) Near-infrared (spectral region: 9000–4000 cm^−1^); (**B1**) mid-infrared (spectral region: 1800–700 cm^−1^) spectra and corresponding model loadings (**A2**,**B2**). Legend: red circles—cotton samples; green squares—PES samples.

Near-infrared and mid-infrared spectra of cotton and PES samples (100%) were used to develop a PLSDA regression model (Figure 2A1,B1, respectively), aiming at sample discrimination according to their composition. 

A clear discrimination of the samples was achieved for both models in the first latent variable (LV1), which encompassed 94.75% (NIR) and 94.54% (MIR) of spectral variability. Also, the infrared spectra (NIR and MIR) of PES samples seemed to present a more dissimilar spectral pattern, a conclusion achieved from their higher dispersion across the scores map.

The percentage of correct sample classifications according to fibre content obtained from the PLSDA model’s confusion matrices were 100% for both near-infrared and mid-infrared spectroscopy (Table S2-Supplementary Materials). Model loadings (of LV1) are represented in Figure 2A2,B2 for near-infrared and mid-infrared spectroscopy, respectively, and allow us to infer the spectral features with the highest impact on sample classification. Higher loadings were observed for wavelengths with higher impact on discrimination. 

**Figure 3:**
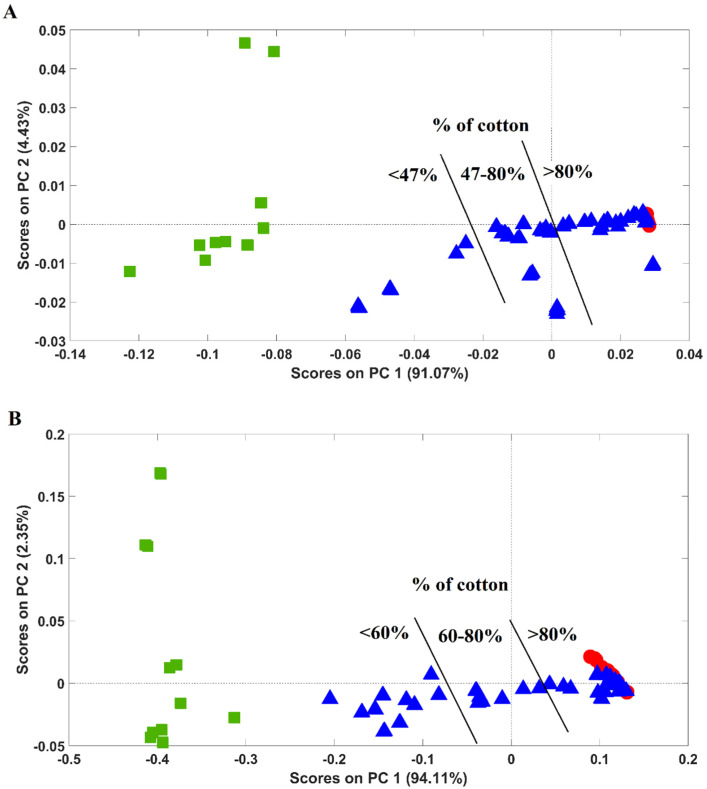
Score plots of the PCA models developed with 100% cotton, 100% PES, and blended samples of cotton and polyester: (**A**) near-infrared (spectral region: 9000–4000 cm^−1^) and (**B**) mid-infrared (spectral region: 1800–700 cm^−1^). Legend: red circles—cotton samples; green squares—PES samples; blue triangles—blended samples.

Two additional PCA models were developed (Figure 3A—near-infrared, and Figure 3B—mid-infrared spectroscopy), including the above-mentioned samples (100% cotton + 100% PES) and blended samples of cotton and PES in different percentages (40 samples containing 37.2–99.7% of cotton). A PLSDA model was not considered since the blended samples are composed of a wide range of cotton/PES percentages, preventing an unequivocal class assignment (it should be noted that PLSDA is a supervised chemometric model requiring sample class assignment before model development).

Globally, for both infrared regions (near- and mid-), cotton and PES samples clustered very similarly to what was observed in the PLSDA model developed solely with 100% cotton and PES samples (unequivocally separated in the first principal component, PC1). Concerning blended samples, these were distributed in the score map between 100% cotton and 100% PES samples, with a few blended samples clustering together with 100% cotton ones.

Blended samples’ distribution was found to be correlated with their cotton/PES content, with the samples in the negative part of PC1 (where 100% PES samples are located) possessing lower percentages of cotton, while samples located in the positive part of the PC1 (where 100% cotton samples were located) displaying higher ones. Blended samples clustering with 100% cotton ones exhibited more than 90% of cotton. 

### 2.3. Cotton Sample Content Estimates

**Figure 4:**
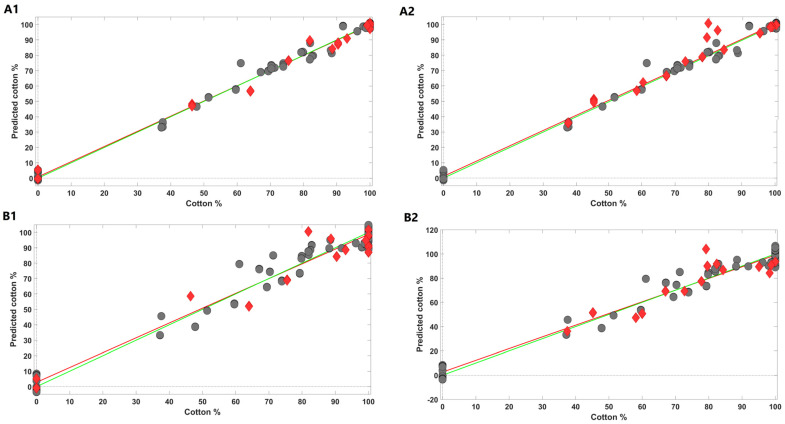
PLS regression results obtained with NIR (**A1**—prediction set 1 and **A2**—prediction set 2) and FTIR spectra (**B1**—prediction set 1 and **B2**—prediction set 2). Legend: grey circles—calibration spectra; red rhombi—prediction spectra.

The samples’ positions in the PLSDA score map were found to have a good correlation with the cotton percentage of each sample for both NIR and MIR data. In this context, PLS regression models were developed and further validated to estimate the cotton percentage of the samples included in this study with NIR and MIR data. The PLS models were developed and optimized according to what is described in the Materials and Methods section (4.4. Data Analysis). The optimum models were further validated with two independent sample sets: Pred 1, containing blended samples of cotton and PES, and Pred 2, comprising samples of cotton, PES, and one additional fibre (linen, viscose, elastane, or polyamide). The plots of the interrelationship between the measured and predicted values are shown in Figure 4 and figures-of-merit obtained from the optimum PLS models are summarized in Table 1. 

Regarding NIR spectra, the highest-performance PLS regression model (lower root mean square of cross-validation) was obtained using a spectral region of 9100–4000 cm^−1^, a number of latent variables of 6, and spectral pre-processing settings snv + SavGol (15, 2, 2). Calibration and cross-validation errors were 3.0% and 3.2%, respectively, while the corresponding determination coefficients were 0.99 for both. The validation of the PLS model with blended samples containing solely cotton and PES (set of samples Pred 1) led to very similar results to those obtained in the calibration step; namely RPSEP = 3.6% and a determination coefficient of 0.99. Also, the RER value was 27.9, meaning that the model possessed a very good potential for quantification proposes. The RPD value was 9.17, indicating excellent model predictions. An additional validation of the PLS model was undertaken using blended samples containing cotton, polyester, and a different fibre (set of samples Pred 2). The RMSEP for this set of samples was higher (7.8%) and the determination coefficient decreased to 0.90, which was somewhat expectable.

With MIR data, the highest-performance PLS model was obtained using a spectral region of 1800–600 cm^−1^, a number of latent variables of 7, and spectral pre-processing settings snv + SavGol (15, 2, 2). Calibration and cross-validation errors were 5.8% and 6.5%, respectively, and the corresponding determination coefficients were 0.97 and 0.96. Regarding prediction set 1, a RMSEP = 6.5 and a determination coefficient of 0.97 were obtained. The RER value obtained was 15.4 and the RPD was 5.08, indicating excellent model predictions. Less promising results were obtained for prediction set 2 (blended samples of cotton, polyester, and an additional fibre): RMSEP = 8.0% and a determination coefficient of 0.82.

## 3. Discussion

The infrared spectral signatures of the cotton and PES samples included in this work were quite consistent with those reported in previous studies [5,22,23,24,25,26,27], which strengthens the relevance of IR spectroscopy for unequivocal sample identification. Besides sample identification, these spectroscopic techniques have been successfully employed for other purposes, which attests to their versatility in the textile industry. Liu and Chang (2024) were able to link different intensities of vibration bands at 956, 1032, and 1500 cm^−1^ to cotton fibre maturity [28]. Parida and colleagues (2024) identified cotton and polyester microfibres collected from the effluent outlet pipe of a washing machine [29]. These authors related differences in intensity plus the presence/absence of some infrared absorption bands to the water temperature used in the washing program. Prajapati et al. also used infrared and Raman spectroscopy to identify and quantify microplastics and microfibres, respectively, in city effluents [30] through their spectral features.

The success of NIR and MIR spectroscopy in textile sample identification and/or discrimination were proved herein, with 100% of correct cotton and polyester sample identification. Studies in the literature on the use of MIR in textile sample discrimination prevent a direct comparison of results as they use fibres other than cotton and polyester in their models. Regarding NIR spectroscopy, more studies aiming at textile sample identification and/or discrimination were encountered in the literature [14,15,16]. Cleve and colleagues (2000) made a successful attempt to discriminate several textile fibres from each other (including cotton and PES). Despite their reported success, the methodology behind sample preparation was quite laborious, including the use of liquid nitrogen to turn the fibres into powder. In the remaining two studies [15,16], a more straightforward analytical approach was utilized; however, Zhou and colleagues reported that only 93% and 92% of PES and cotton samples, respectively, were correctly discriminated. Furthermore, all these articles included a single fibre presentation (most of them woven fabric) and did not account for the diversity of samples considered in our study (raw, yarn, and knitted and woven fabric). NIR spectroscopy was also reported as a very useful technique to sort textile samples according to their composition [31,32,33], but this protocol has never been tested for blended samples.

Regarding composition estimates, both techniques (NIR and MIR spectroscopies) appear to have good potential for the estimation of cotton content in textile samples, with the results obtained using NIR spectroscopy being slightly better. It is of note that the PLS models developed for both techniques were quite well validated (RMSEP = 3.6% and 6.5% for NIR and MIR data, respectively) for prediction set 1. The results became inferior when the validation was undertaken with prediction set 2, since these samples contained additional fibres that were not included in the calibration step (linen, viscose, elastane, and polyamide). This lower performance is a recognized drawback of such calibration models. To the best of our knowledge, no other studies have used MIR spectroscopy for composition estimation, preventing a comparison of our study with those in the literature. However, NIR spectroscopy has already been used in prior research [17,18,19]. The results obtained in the present work are consistent with the ones reported in those studies. However, it should be stressed that the published results regard only one type of textile sample (knitted and/or woven fabric), never considering distinct sample presentations in the same model. Moreover, previously published studies do not validate the developed regression models with blended samples containing additional fibres on top of those included in the calibration sets.

## 4. Materials and Methods

### 4.1. Textile Samples

**Figure 5:**
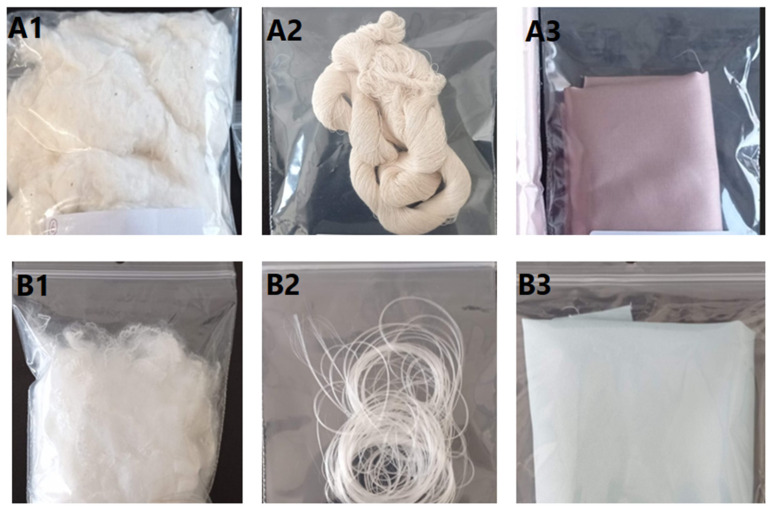
Pictures of 100% cotton (**A1**—raw; **A2**—yarn; **A3**—fabric) and 100% polyester (**B1**—raw; **B2**—yarn; **B3**—fabric) samples.

The textile samples used in this work (n = 84) were provided by CITEVE in the context of the BE@T project (Table 2). Pictures of 100% cotton (raw, yarn, fabric) and 100% polyester samples (raw, yarn, fabric) are shown in Figure 5 for illustration purposes. Samples included raw fabric (n = 14), yarn (n = 39), knitted fabric (n = 14), and woven fabric (n = 17) samples and were of different composition: 100% cotton (n = 26); 100% polyester (n = 10); cotton/polyester blend (n = 40); and cotton/polyester/additional fibre (n = 8). Further details on the samples can be found in Table S1-Supplementary Materials.

### 4.2. Near-Infrared Spectrum Acquisition

The NIR spectra of the textile samples were obtained on a Fourier transform near-infrared spectrometer (spectrum two, Perkin Elmer) with an indium–gallium–arsenide (InGaAs) detector in diffuse reflectance mode. Each spectrum resulted from the average of 100 scans with a resolution of 8 cm^−1^ within the wavenumber interval of 10,000 to 4000 cm^−1^. Five spectra (instrumental replicates) per sample were collected in five different sample spots and used for further analysis. The background was acquired between each sample using a reference material (Teflon). 

### 4.3. Mid-Infrared Spectrum Acquisition

The MIR spectra of the textile samples were obtained on a Fourier transform Perkin Elmer Spectrum BX FTIR System spectrophotometer (Perkin Elmer, Shelton, CT, USA) with a DTGS detector. The spectra were acquired in diffuse reflectance mode through a PIKE Technologies Gladi attenuated total reflectance (ATR) accessory (Pike Technologies, Madison, WI, USA) within the wavenumber interval of 4000 to 600 cm^−1^, with a resolution of 4 cm^−1^ and 32 scan co-additions. Each sample was placed on top of the ATR crystal, and constant pressure was applied. Five spectra (instrumental replicates) per sample were acquired in five different sample spots. The ATR crystal was cleaned, and a background was acquired between each sample.

### 4.4. Data Analysis

Principal component analysis (PCA) [34], partial least squares discriminant analysis (PLSDA) [35], and partial least squares (PLS) [36] were the chemometric models selected to analyze the near-infrared and mid-infrared data. Before the application of each chemometric model, the spectra were consistently pre-processed and further mean-centred. The pre-processing methods were standard normal variate (snv) [36] and the Savitzky–Golay (SavGol) filter [37], whose parameters (x—filter width; y—polynomial order; and z—derivative order) were optimized during the PLS model’s development.

The PLSDA models were developed to evaluate the ability of the spectroscopic techniques to discriminate between pure cotton (100%) and polyester (100%) samples and further obtain the corresponding confusion matrices with the percentage of correctly discriminated samples. PLSDA is a supervised method based on the PLS regression method. In PLSDA models, each sample spectrum (*x_i_*) is assigned a vector of zeros, with a value of one at the position corresponding to its class (*y_i,_* cotton), in such a way that categorical variable values (*y_i_*) can be predicted for samples of unknown composition (cotton or polyester). Model loadings and the corresponding scores were obtained by sequentially extracting the components or latent variables (LVs) from matrices *X* (spectrum) and *Y* (matrix codifying composition). In PLSDA, a probability value for each assignment is estimated for each sample. The number of latent variables (LVs) was optimized using the leave-one-sample-out cross-validation procedure in order to prevent over-fitting, considering only 70% of the available data (randomly selected). After the optimization of the number of LVs, the model was tested on the remaining 30% samples to assess the proportion (%) of correct predictions for each class [38,39] given in the form of a confusion matrix. 

The PCA models were generated to evaluate the clusterization of pure (100% cotton and 100% polyester) and blended (cotton + polyester) samples. Lastly, the PLS models were utilized to evaluate the potential of the techniques to predict the cotton percentage in blended (cotton + polyester) samples. PLS models were optimized regarding the number of latent variables (LVs) (2 to 10 LVs), the spectral region to be used, and the SavGol filter parameters (filter width: 9, 12, and 15 points; polynomial order: 2; derivative order: 1st and 2nd). The spectral regions tested (alone and or in combination) are specified in Figure S1A-Supplementary Materials for the near-infrared (31 combinations) and S1B-Supplementary Materials for mid-infrared (15 combinations) spectra. 

The following parameters were used to evaluate the accuracy of the PLS models: root mean square error of calibration, cross-validation, and prediction (RMSEC, RMSECV, and RMSEP, respectively); prediction determination coefficient (R2P); and range error ratio (RER). The RMSEC, RMSECV, and RMSEP were estimated according to Equation (1):

(1) $$RMSE=\sqrt{\frac{\sum_{i=1}^{N}{\hat{(Yi}-Yi)}^{2}}{N}}$$

In Equation (1), N is the number of samples, Yi is the experimental measurement result for sample i, and $\hat{Y}i$ is the corresponding value obtained for the calibration (RMSEC), the cross-validation (RMSECV), and the prediction set (RMSEP) for each sample. The experimental values and the estimates obtained for the prediction sets were compared through Pearson’s correlation coefficient. The RER parameter was also used to evaluate the accuracy of the predictive model and it gives the ratio between the ranges of the parameter values in the prediction set with the respective RMSEP for set of samples Pred 1. It was calculated according to Equation (2):

(2) $$RER=\frac{\left(Y_{max}-Y_{min}\right)}{RMSEP}$$

In Equation (2), $Y_{max}$ and $Y_{min}$ are the maximum and minimum values in the prediction set. RER values above 10 indicate good predictive models and those above 15 are good for calibration and quantification determinations according to the guidelines for near-infrared model development and maintenance [40]. 

The residual prediction deviation (RPD) value was also determined to evaluate the PLS models’ performance.

In Equation (3), std is standard deviation of the data. RPD values < 1.0 indicate very poor model predictions and their use is not recommended; RPD between 1.0 and 1.4 indicate poor model predictions; RPD between 1.4 and 1.8 indicate fair predictions; between 1.8 and 2.0 indicate good predictions; between 2.0 and 2.5 indicate very good predictions; and RPD > 2.5 indicate excellent model predictions [41].

(3) $$RPD=\frac{std}{RMSEP}$$

Matlab version R2023a (MathWorks, Natick, MA, USA) and the PLS Toolbox version 9.2.1 (Eigenvector Research Incorporated, Manson, WA, USA) were used for all the calculations. 

## Figures and Tables

**Table 1:** Figures-of-merit obtained from de optimum PLS models developed with NIR and MIR spectra.

Sample Composition	Set	Figures-of-Merit	Near-IR	Mid-IR
Cotton//PES	Cal	RMSEC	3.0	5.8
		R^2^C	0.99	0.97
		RMSECV	3.2	6.5
		R^2^CV	0.99	0.96
	Pred 1	RMSEP	3.6	6.5
		R^2^P	0.99	0.97
		RER	27.9	15.4
		RPD	9.17	5.08
Cotton//PES//Other	Pred 2	RMSEP	7.8	8.0
		R^2^P	0.90	0.82

RMSEC—root mean square error of calibration; RMSECV—root mean square error of cross-validation; RMSEP—root mean square error of prediction; R^2^C—determination coefficient of calibration; R^2^CV—determination coefficient of cross-validation; R^2^P—determination coefficient of prediction; RER—range error ratio; RPD—residual prediction deviation.

**Table 2:** Textile samples included in this work.

Nº Samples	Type of Sample (Nº)	Composition (% Range)
26	Raw (7) Yarn (12) Woven fabric (7)	100% cotton
10	Raw (3) Yarn (3) Knitted fabric (3) Woven fabric (1)	100% polyester
40	Raw (3) Yarn (19) Knitted fabric (11) Woven fabric (7)	99.7–37.2% cotton 0.3–62.8% polyester
8	Raw (1) Yarn (5) Woven fabric (2)	98.3–37.5% cotton 54.3–0.3% polyester 19.8–0.4% other fibre (linen, viscose, elastane, and polyamide)

## Data Availability

No new data was created.

## Supplementary Materials

The following supporting information can be downloaded at: https://www.mdpi.com/article/10.3390/molecules29153667/s1, Figure S1: Typical near-infrared (A) and mid-infrared (B) spectra of cotton illustrating the spectral regions used (alone and combined) for chemometric model optimizations; Table S1: Detailed information about the textile samples included in this work; Table S2: Confusion matrices obtained from the PLSDA regression models developed with the near-infrared and mid-infrared spectra of 100% cotton and 100% polyester samples.
