# Supplementary material for: Discrimination and Quantification of Cotton and Polyester Textile Samples Using Near-Infrared and Mid-Infrared Spectroscopies
Source: Molecules. 2024 Aug 2;29(15):3667. doi: 10.3390/molecules29153667 (PMC11313922; doi:10.3390/molecules29153667)
Supplement: Supplementary file 1 [file molecules-29-03667-s001.zip › molecules-3079263-supplementary.pdf]

Supplementary Material

Cotton and polyester textile samples deciphered by near and mid-infrared spectroscopy: discrimination and quantification.

Maria Luís Paz, Clara Sousa*

Universidade Católica Portuguesa, CBQF – Centro de Biotecnologia e Química Fina – Laboratório Associado, Escola Superior de Biotecnologia, Rua Diogo Botelho 1327, 4169-005 Porto, Portugal

* corresponding author: cssousa@ucp.pt

Table S1. Detailed information about the textile samples included in this work

Sample	Set	Presentation	Fibers	Composition (cotton // PES // Other) (%)
1	Cal	Raw	Cotton	100 // 0 // 0
2	Cal	Raw	Cotton	100 // 0 // 0
3	Pred 1	Raw	Cotton	100 // 0 // 0
4	Pred 1	Raw	Cotton	100 // 0 // 0
5	Cal	Raw	Cotton	100 // 0 // 0
6	Cal	Raw	Cotton	100 // 0 // 0
7	Cal	Raw	Cotton	100 // 0 // 0
25	Cal	Yarn	Cotton	100 // 0 // 0
36	Pred 1	Woven fabric	Cotton	100 // 0 // 0
37	Cal	Woven fabric	Cotton	100 // 0 // 0
38	Cal	Woven fabric	Cotton	100 // 0 // 0
47	Cal	Yarn	Cotton	100 // 0 // 0
48	Cal	Yarn	Cotton	100 // 0 // 0
49	Pred 1	Yarn	Cotton	100 // 0 // 0
50	Pred 1	Yarn	Cotton	100 // 0 // 0
51	Pred 2	Yarn	Cotton	100 // 0 // 0
52	Cal	Yarn	Cotton	100 // 0 // 0
53	Cal	Yarn	Cotton	100 // 0 // 0
54	Cal	Yarn	Cotton	100 // 0 // 0
61	Cal	Yarn	Cotton	100 // 0 // 0
66	Pred 1	Woven fabric	Cotton	100 // 0 // 0
67	Cal	Woven fabric	Cotton	100 // 0 // 0
68	Cal	Woven fabric	Cotton	100 // 0 // 0
69	Cal	Woven fabric	Cotton	100 // 0 // 0
72	Cal	Yarn	Cotton	100 // 0 // 0

73	Cal	Yarn	Cotton	100 // 0 // 0
56	Pred 1	Knitted fabric	PES	0 // 100 // 0
57	Cal	Yarn	PES	0 // 100 // 0
58	Cal	Yarn	PES	0 // 100 // 0
60	Cal	Raw	PES	0 // 100 // 0
74	Pred 1	Raw	PES	0 // 100 // 0
75	Cal	Raw	PES	0 // 100 // 0
76	Cal	Yarn	PES	0 // 100 // 0
79	Cal	Knitted fabric (cyan blue)	PES	0 // 100 // 0
80	Cal	Knitted fabric (lilac)	PES	0 // 100 // 0
81	Cal	Woven fabric	PES	0 // 100 // 0
F1	Cal	Yarn	Cotton // PES	99.7 // 0.3 // 0
F2	Pred 1	Yarn	Cotton // PES	99.2 // 0.8 // 0
F71	Cal	Raw	Cotton // PES	99.1 // 0.9 // 0
F4	Pred 2	Raw	Cotton // PES	98.8 // 1.2 // 0
F72	Cal	Yarn	Cotton // PES	98.8 // 1.2 // 0
F5	Cal	Woven fabric	Cotton // PES	98 // 2 // 0
F7	Cal	Yarn	Cotton // PES	96.2 // 3.8 // 0
F8	Pred 1	Yarn	Cotton // PES	93.1 // 6.9 // 0
F74	Cal	Woven fabric	Cotton // PES	91.9 // 8.1 // 0
F75	Pred 1	Raw	Cotton // PES	90.4 // 9.6 // 0
F10	Pred 1	Knitted fabric	Cotton // PES	88.7 // 11.3 // 0
F11	Cal	Woven fabric	Cotton // PES	88.5 // 11.5 // 0
F12	Cal	Knitted fabric	Cotton // PES	88.2 // 11.8 // 0
F13	Pred 2	Yarn	Cotton // PES	84.3 // 15.7 // 0
F76	Cal	Knitted fabric	Cotton // PES	82.9 // 17.1 // 0
F77	Cal	Woven fabric	Cotton // PES	82.5 //17.5 // 0
F78	Cal	Knitted fabric	Cotton // PES	82 // 18 // 0
F79	Cal	Knitted fabric	Cotton // PES	81.9 // 18.1 // 0
F80	Pred 1	Knitted fabric	Cotton // PES	81.9 // 18.1 // 0

F81	Cal	Knitted fabric	Cotton // PES	79.9 // 20.1 // 0
F82	Cal	Knitted fabric	Cotton // PES	79.8 // 20.2 // 0
F83	Cal	Knitted fabric	Cotton // PES	79.3 // 20.7 // 0
F84	Pred 2	Yarn	Cotton // PES	77.8 // 22.2 // 0
F86	Pred 1	Yarn	Cotton // PES	75.5 // 24.5 // 0
F87	Cal	Yarn	Cotton // PES	73.9 // 26.1 // 0
F88	Cal	Knitted fabric	Cotton // PES	71.3 // 28.7 // 0
F89	Cal	Yarn	Cotton // PES	70.3 // 29.7 // 0
F19	Cal	Yarn	Cotton // PES	69.4 // 30.6 // 0
F90	Cal	Yarn	Cotton // PES	67.1 // 32.9 // 0
F91	Pred 2	Yarn	Cotton // PES	67 // 33 // 0
F92	Pred 1	Woven fabric	Cotton // PES	64 // 36 // 0
F93	Cal	Knitted fabric	Cotton // PES	61.1 // 38.9 // 0
F94	Cal	Yarn	Cotton // PES	59.6 // 40.4 // 0
F95	Pred 2	Yarn	Cotton // PES	58.1 // 41.9 // 0
F21	Cal	Yarn	Cotton // PES	51.4 // 48.6 // 0
F98	Cal	Yarn	Cotton // PES	47.8 // 52.2 // 0
F101	Pred 1	Yarn	Cotton // PES	46.4 // 53.6 // 0
F102	Pred 2	Yarn	Cotton // PES	45.2 // 54.8 // 0
F103	Cal	Woven fabric	Cotton // PES	37.6 // 62.4 // 0
F104	Cal	Woven fabric	Cotton // PES	37.2 // 62.8 // 0
F41	Pred 2	Yarn	Cotton // PES // Linen	82.4 // 2.5 // 15.1
F109	Pred 2	Yarn	Cotton // PES // Linen	79.6 // 0.6 // 19.8
F108	Pred 2	Raw	Cotton // PES // Viscose	98.3 // 0.3 // 1.4
F107	Pred 2	Yarn	Cotton // PES // Viscose	37.5 // 54.3 // 8.2
F48	Pred 2	Woven fabric	Cotton // PES // Viscose	79.2 // 18.7 // 2.1
F43	Pred 2	Yarn	Cotton // PES // Elastane	95.1 // 0.7 // 4.2
F194	Pred 2	Fabric	Cotton // PES // Elastane	60 // 37.4 // 2.6
F113	Pred 2	Woven fabric	Cotton // PES // Polyamide	72.7 // 26.9 // 0.4

Table S2. Confusion matrices obtained from the PLSDA regression models developed with nir and mid-infrared spectra of 100% cotton and 100% polyester samples.

	**Near IR prediction**				**Mid IR prediction**		
**Sample class**	**Cotton**	**Polyester**		**Sample class**	**Cotton**	**Polyester**	
**Cotton**	75	0		**Cotton**	75	0	
**Polyester**	0	25		**Polyester**	0	25	
Total % of correct classification			100	Total % of correct classification			100

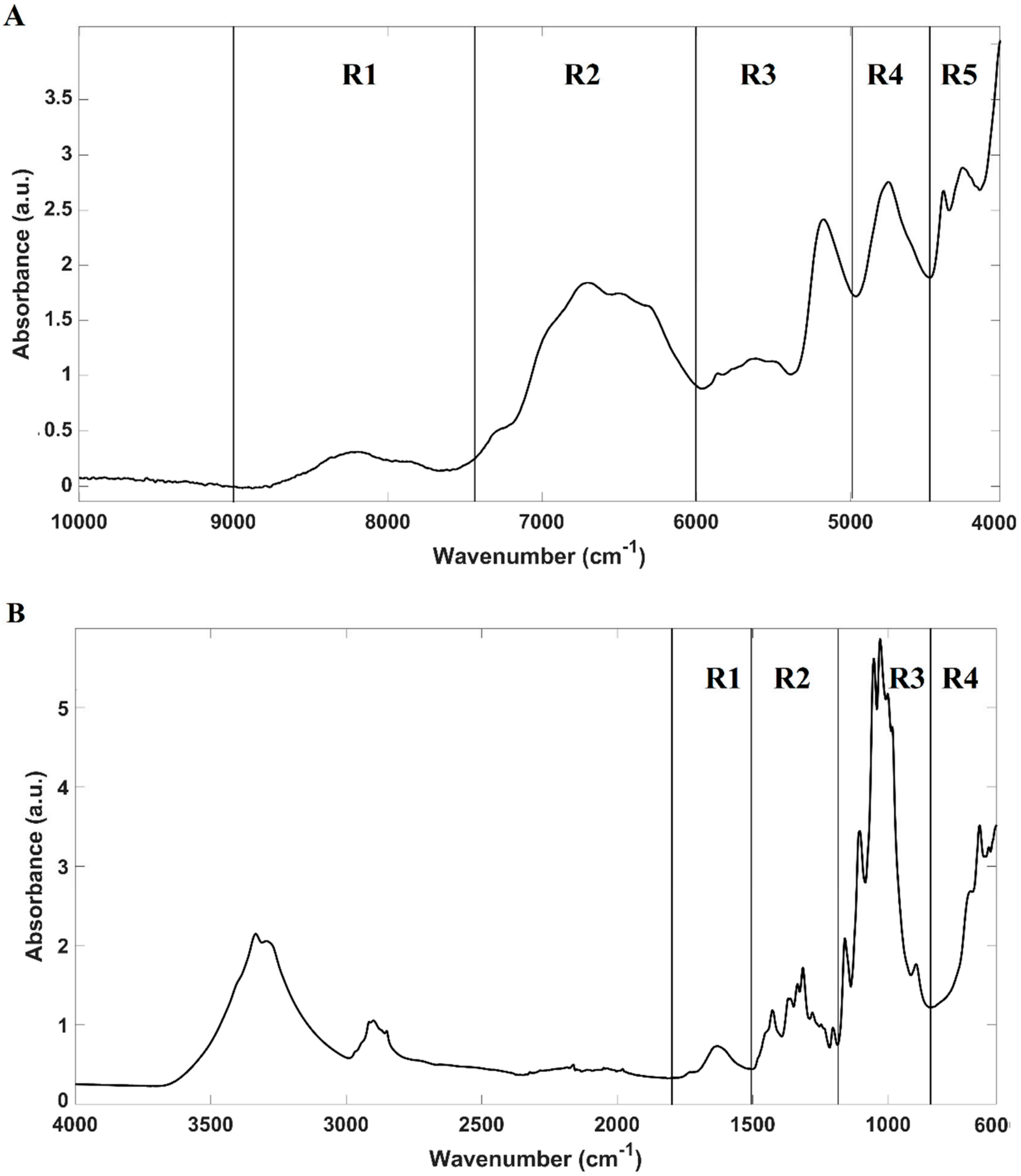

Figure S1. Typical near (A) and mid-infrared (B) spectra of cotton illustrating the spectral regions used (alone and combined) for the chemometric models optimizations.
